# Clinical Reformation in the Age of Artificial Intelligence: Safeguarding the Ethical Centre of Medicine

**DOI:** 10.1016/j.mcpdig.2025.100310

**Published:** 2025-12-05

**Authors:** Ian Io Lei, Wojciech Marlicz, Ramesh P. Arasaradnam, Anastasios Koulaouzidis

**Affiliations:** a Institute of Precision Diagnostics & Translational Medicine, University Hospital of Coventry and Warwickshire, Coventry, UK; b Warwick Medical School, University of Warwick, Coventry, UK; c Department of Gastroenterology, Pomeranian Medical University in Szczecin, Poland; d Leicester Cancer Centre, University of Leicester, UK; e Department of Clinical Research, University of Southern Denmark, Denmark; f Department of Surgery, Odense University Hospital, Denmark

Artificial intelligence (AI) is transforming health care, with a projected market value of USD 187 billion by 2030.^1^ However, biases and inequitable access threaten patient outcomes.^2^ Rapid adoption risks amplifying disparities if not guided by ethical frameworks. This rapid diffusion coincides with systemic fragility, characterized by clinician burnout, strained resources, and widening inequities. Artificial intelligence is extolled as a solution for efficiency and precision, but this risks neglecting a deeper question: what kind of medicine will it create?

Integrating AI is beyond a technical challenge; it is a socio-technical one. Success depends on a superstructure of ethics, governance, culture, and human-centered design. We contend that AI must be embedded not only for accuracy but also for morality, ie, safeguarding trust, interpretation, and human connection. Without deliberate design, care risks being reduced to prediction and optimization, eroding the relational essence of healing. A clinical reformation is therefore required to align AI with medicine’s enduring purpose.

This perspective proposes a new conceptual framework, Predictive, Assistive, Narrative, Equity-oriented, Ethical, and Augmentative (PANEEA), to guide the ethical integration of AI into clinical medicine. Each component of PANEEA represents a design and evaluative principle that places the moral and relational core of medicine at the center of technological change. Unlike technical reviews or implementation roadmaps, this paper offers a bioethics-driven perspective grounded in narrative ethics, global health equity, and professional integrity.

## Evidence Gaps and the Need for Hybrid Research

Artificial intelligence promises consistency and predictive accuracy, however, the evidence base is uneven. A 2020 systematic review reported high bias and limited external validity in deep learning AI studies comparing AI to clinicians,^2^ whereas a 2025 meta-analysis confirmed persistent design flaws and poor generalizability.^3^ A 2024 scoping review found that only 30% of AI randomized controlled trials adequately reported demographic characteristics, equity, or applicability.^4^ Overall, only 5% of AI studies are randomized controlled trials. This weak foundation risks narrowing medicine: patient narratives are reduced to algorithmic signals, and clinicians are recast as machine supervisors rather than interpreters of human experience. Empathy and moral discernment cannot be automated and must remain design priorities. Simulation-based research, in silico trials, and adaptive algorithms accelerate innovation, but reporting standards remain poor. Ophthalmology trials, for example, often omit CONSORT-AI requirements such as error handling,^5^ whereas TRIPOD-AI evaluations highlight underreporting of calibration and bias.^6^ A comprehensive 2024 systematic review by Kolbinger et al^7^ found that despite a proliferation of AI studies in medicine, most remain in preclinical or translational phases, with few progressing to robust clinical validation. The majority of AI reporting guidelines (26 were reviewed) lack standardized methodology, with over 40% relying on nonconsensus, expert-led processes.^7^ Moreover, reporting and quality appraisal frameworks must evolve to address the distinct epistemological and operational complexities introduced by machine learning systems, particularly in dynamic or low-resource clinical settings where AI tools are most often proposed but least validated. Although foundational instruments like TRIPOD-AI and CONSORT-AI provide general guidance, recent developments such as The Prediction model Risk Of Bias ASsessment Tool for Artificial Intelligence^8^ and Quality Assessment of Diagnostic Accuracy Studies for Artificial Intelligence,^9^ represent a new generation of tools designed specifically for the appraisal of AI-based prediction and diagnostic models.

Hybrid models, combining AI’s predictive power with clinician expertise, can mitigate bias, as shown in China’s AI-driven diagnostics.^10^ We extend this principle to research, proposing a hybrid model: simulation-led exploration paired with human-validated trials that assess not only accuracy but also trust, adherence, recovery, quality of life, and equity. This model suits current narrow AI systems, which remain task-specific and bounded. As AI advances toward broader systems that integrate notes, laboratory results, imaging, and genomics for personalized advice, stronger multistakeholder frameworks will be needed to balance innovation with safety, equity, and compassionate, expert-led care. A 2025 review reinforces the need for outcomes beyond accuracy to prevent the widening of health disparities.^10^ Regulators and journals should therefore mandate the open sharing of data, transparent code, and the reporting of demographic characteristics.

### Reshaping Clinical Roles and Training

Artificial intelligence is reshaping clinical roles, with digital triage systems and chatbots emerging as primary points of contact in care, while precision medicine increasingly relies on AI for risk stratification. Medical curricula must therefore embed probabilistic reasoning, recognition of algorithmic bias, AI ethics, emotional intelligence, and narrative competence. The European Union Artificial Intelligence Act establishes governance for the deployment of AI.^11^ Within this, clinicians will continue to lead through interpretive skill and moral judgment. This is further supported by comprehensive AI literacy programs and strengthened by robust incident reporting mechanisms.

However, a 2023 study reported that chatbots were rated as more empathetic than physicians in responding to online patient queries, although this finding was confined to social media contexts.^12^ This highlights AI’s potential to improve communication while warning of the risk that clinicians may relinquish relational roles without sufficient training. A 2025 review advocates for embedding AI literacy into medical curricula.^13^ For instance, Stanford’s AI in Medicine course equips clinicians with skills in algorithmic interpretation, preparing them for collaborative practice. Yet accountability and the act of bearing witness must remain inherently human responsibilities.

### Equity, the Global Digital Divide, and Intersectional Considerations

Artificial intelligence development is concentrated within high-income countries, assuming infrastructure is absent in low-income and middle-income countries. That risks deepening inequities. The Rwanda Artificial Intelligence for Diabetic Retinopathy Screening trial in Rwanda increased diabetic retinopathy screening uptake,^14^ and India’s AI- Driven Diabetic Retinopathy Screening System tool reported high sensitivity across diverse populations.^15^ Federated learning can extend such benefits.

Artificial inelligence for tuberculosis screening in South Africa achieved 90% sensitivity, but only 15% of LMIC hospitals have AI infrastructure, highlighting the digital divide. Equity requires epistemic justice, including validation of marginalized voices (eg, indigenous health paradigms) within the AI design and evaluation. Systematic reporting by race, ethnicity, gender, age, disability, and sexual orientation is essential to counter bias.^16^ A 2025 WHO initiative emphasizes the engagement of low-income and middle-income countries in AI governance.^17^ Practical challenges, such as the frequent misclassification of transgender patient data, illustrate the need for inclusive and context-sensitive design.

### Restoring Narrative to Clinical Documentation

Artificial intelligence tools purport to improve clinical documentation by structuring data, generating narratives, and reducing errors by 19%-92% mainly in documentation time.^18^ Electronic health records often prioritize payment schedules and time-to-treat targets over patient outcomes. Artificial intelligence risks exacerbating this tendency through templated notes; however, it also offers a remedy if co-designed to capture nuance, uncertainty, and temporality. Clinical records must remain ethical spaces that capture human frailty and healing. A forward-looking approach is to draw on the digital humanities, a field that has long grappled with questions of narrative and computation, to explore how computational tools can generate richer, more dynamic forms of clinical storytelling than were possible in the analog era.^19^ AI-enhanced electronic health records systems, such as ChatEHR,^20^ should enable clinicians to query records conversationally, restoring patient centered narratives while ensuring integrity.

### Principles for a Moral AI-Enabled Medicine

Herein, we propose the PANEEA Principles, a normative framework for ethically grounded health care and research: patient autonomy and involvement (P), accountability and oversight (A), narrative integrity (N), equity in global health (E), education for moral agency (E), and humanistic augmentation (A). These principles strike a balance between technological innovation and human values, advancing scientific rigor, justice, sustainability, and moral responsibility. PANEEA serves as both a guide and a call to action, ensuring progress in health is measured by integrity, dignity, and equity.

#### Patient Autonomy and Involvement

Respect for patient autonomy remains foundational. AI-enabled care must include informed consent and active patient involvement in the design and governance.^17^ For example, patient advisory boards guide development to align with lived experiences. Metrics include universal consent compliance and meaningful patient involvement in at least 50% of AI projects.

#### Accountability and Oversight

Robust governance prevents harm and upholds standards, including transparency in datasets, models, and decisions through explainable AI (eg, heatmaps). Independent ethics boards and postdeployment monitoring are essential.^5^ For instance, regulatory audits identify bias. Measures include % of deployments under oversight and error correction rates.

#### Narrative Integrity

Documentation must preserve patient stories and continuity. Co-designing AI tools captures nuance and temporality; narrative audits safeguard completeness.^21^ For example, AI-assisted notes reflect illness trajectories. Metrics include narrative completeness scores.

#### Equity in Global Health

AI development in high-income settings risks inequities; federated learning and infrastructure-light tools ensure validity across populations, including environmental impact assessments for sustainability.^17^^,^^22^ An example is Rwanda’s retinopathy screening. Indicators include LMIC adoption rates and reductions in dataset bias.

#### Education for Moral Agency

Clinicians need ethical and technical literacy, with curricula on bias detection, contextual reasoning, and sensitivities.^13^ Examples include postgraduate programs embedding AI ethics. Outcomes include the proportion of individuals achieving AI literacy, with the goal of universal competency by 2030.

#### Humanistic Augmentation

AI enhances empathy, not erodes it, through hybrid workflows allowing more interpersonal time.^4^^,^^12^ Examples include supervised chatbots. Success metrics extend beyond accuracy to patient satisfaction and clinician relational care time one-third or more.

## Conclusion

Medicine’s future demands the synthesis of technological innovation with the moral traditions that safeguard human dignity. Artificial intelligence has the potential to enhance care, but only if anchored in equity, presence, and discernment. Its integration must prioritize patient outcomes over metrics, restore narrative integrity to clinical practice, and prepare clinicians to act as ethical leaders in an era of accelerating automation. If deliberately shaped in this way, AI will not displace medicine’s purpose but deepen it, ensuring that empathy, social interpretation, and healing remain at the heart of care.

## Potntial Competing Interests

Dr Koulouzidis is a cofounder and shareholder of AJM Medicaps. He is consultant for Jinshan and has been director and shareholoder of iCERV. He has received travel support (Jinshan, DiagMed Healthcare Ltd, Aquilant), research support (grant) from ESGE/Given Imaging Ltd. and (material) IntroMedic/SynMed; lecture honoraria (Jinshan, Medtronic); and participation in advisory board meetings for Tillots, ANKON, Dr Falk Pharma UK, Jinshan. The other authors report no competing interests.
